# Lipid index changes in the blood serum of patients with hyperplastic and early neoplastic lesions in the ovaries

**DOI:** 10.1186/s13048-014-0090-6

**Published:** 2014-09-27

**Authors:** Mikołaj Karmowski, Krzysztof A Sobiech, Jacek Majda, Piotr Rubisz, Stanisław Han, Andrzej Karmowski

**Affiliations:** First Department of Gynecology and Obstetrics, Wrocław Medical University, Wrocław, Poland; Department of Human Biology, University School of Physical Education in Wrocław, Wrocław, Poland; Department of Laboratory Diagnostics, Fourth Military Hospital, Wrocław, Poland; Hasco-Lek Pharmaceutical Production Company S.A., Wrocław, Poland

**Keywords:** Lipid index, Ovarian neoplasm

## Abstract

**Background:**

The authors used the lipid index (*WL*) to monitor lipid changes before and after surgery. The surgical operation performed was the simultaneous enucleation of a cystic tumor of the hilum ovarii in its entirety (with diagnosis of a simple cyst or teratoma adultum) in groups of 20 patients.

**Objectives:**

To compare the lipid index *WL* in the blood serum of patients undergoing surgery treatment at the following times: before and 7 days after surgery, and 6 and 12 months after surgery.

**Material and methods:**

The research material was the blood serum of women aged about 24 years. The authors divided the patients into 3 groups: two groups of 20 women and a control group. The concentrations of the lipid parameters were measured and the lipid index *WL* was calculated.

**Results:**

Statistically significant differences were found between the lipid index of serum from patients with diagnosed ovarian neoplasms and the index of serum from healthy subjects; differences were demonstrated in the postoperative period, particularly 6 and 12 months after surgery.

**Conclusions:**

The lipid index *WL* proved useful in diagnosing ovarian neoplasm (simple cysts and teratoma adultum) and in monitoring the postoperative period.

## Background

Since 2002, the lipid index has been successfully used in oncogynecological diagnostics. The index is calculated using the concentrations of HDL and LDL lipoprotein, apolipoprotein A1 and B, and triglycerides (TG) [1,2].

The usefulness of this marker has been demonstrated in monitoring changes in lipid metabolism in perimenopausal women undergoing gynecological surgery, as well as in the assessment of hormone replacement therapy [3–5].

Based on these results, it was decided that the objective of the present study would be to determine the diagnostic role of this indicator in the blood serum of women undergoing surgery due to neoplastic hyperplasia within the ovary and parovarian mesonephritic structures. These procedures were performed due to a diagnosis of teratoma: adultum teratoma [6–8] or a simple cyst [9–11].

The motivation to undertake this research lies in the fact that the authors have found no comprehensive publication in the available literature describing the lipid index in gynecological oncology.

## Material and methods

The study included three groups of women, each of up to 20 people.

Group I contained healthy women with an average age of 24.3 years. The criteria for inclusion in Group I were as follows:

- no pathological findings in gynecological examination;
- normal values of urine analyses, blood counts, and diagnostic enzymology;
- age between 18 and 28 years; and
- BMI between 19.5 and 26 kg/m^2^.

The exclusion criterion was a history of liver, kidney, or bile-duct disease.

Patients in Group II were operated on because of neoplastic lesions within the ovarian and parovarian structures (appendages). Histopathological examination showed benign hyperplasia of the simple cyst type was found. The following are the criteria for inclusion into the group:

- age between 18 and 28 years;
- tumor size between 5 and 22 cm, as determined by means of gynecological examination and confirmed by ultrasound examination;
- histopathological diagnosis.

The patients in Group III were operated on because of neoplastic lesions within the appendages (ovary, paroophoron, epoophoron, or other mesonephritic structures). Postoperative histopathological examination revealed a mature teratoma. The indication for surgery in both Groups II and III was tumor size of 5–20 cm in diameter, determined by means of gynecological examination and confirmed by ultrasound examination.

The range of performed operations included enucleation of the cyst in one piece, preserving oncological asepsis and followed by intraoperative pathology consultation.

Biochemical measurements were performed prior to surgery (A), 7 days after surgery (B), and 6 and 12 months after surgery (C and D, respectively).

The research material was blood taken from the basilic vein. The samples obtained from blood serum were analyzed by assessing the concentration of:

- apolipoprotein A and B using immunoturbidimetric method with the Orion Diagnostica reagent on a Technicon RA 1000 (USA) analyzer;
- triglycerides (TG) using the enzymatic colorimetric method;
- lipoprotein HDL cholesterol by precipitation; and
- total cholesterol (TCH) using the enzymatic oxidase method.

The lipoprotein LDL cholesterol level was calculated using the Friedewald formula.

Given the lipid metabolism parameters, the lipid index *WL* was calculated using the formula [1,2]:

$$ WL=\frac{\left(HDL+\frac{TG}{6}\right)*\frac{ApoA1}{30}*10}{\left(LDL+\frac{TG}{5}\right)*\frac{ApoB}{20}} $$

The obtained results are presented as arithmetic means and standard deviations, which were statistically analyzed using Student’s *t*-test and adjusted with the Bonferroni correction, whose significance level for the studied index was 0.05/5 = 0.01.

The study was performed and financed under grant No. 500/03 from Wrocław Medical University, Poland.

## Results

Table 1 presents the data on the lipid index *WL* in the blood serum from the test groups. The results of the statistical analysis are shown in Table 2. It is clear from the data that there was a statistically significant decrease in this parameter in both Groups II and III, compared to the control Group I (p < 0.001). After surgery, Groups II and III showed the highest statistically significant differences between times A and D (p < 0.001). Other results for these two groups between the different time points were highly significant (p < 0.01 and p < 0.001), except for time points A and B, that is, before and 7 days after surgery.

**Table 1:** **Lipid index** ***WL*** **in blood serum in the study groups**

		**Lipid index WL**
**Group I**		**9.99 ± 0.85**
Group II (simple cyst)	A. Before surgery	3.16 ± 0.41
	B. 7 days after surgery	3.19 ± 0.40
	C. 6 months after surgery	4.41 ± 0.60
	D. 12 months after surgery	5.25 ± 0.35
Group III (teratoma adultum)	A. Before surgery	5.94 ± 0.44
	B. 7 days after surgery	5.98 ± 0.48
	C. 6 months after surgery	6.40 ± 0.46
	D. 12 months after surgery	8.51 ± 0.69

**Table 2:** **Statistical analysis**

**Groups**	**P value**
I: II A	0.001
I: III A	0.001
II A: II B	NS
II A: II C	0.001
II A: II D	0.001
II B: II C	0.001
II B: II D	0.001
II C : II D	0.001
III A: III B	NS
III A: III C	0.01
III A: III D	0.001
III B: III C	0.01
III B: III D	0.001
III C: III D	0.001

**Figure 1:**
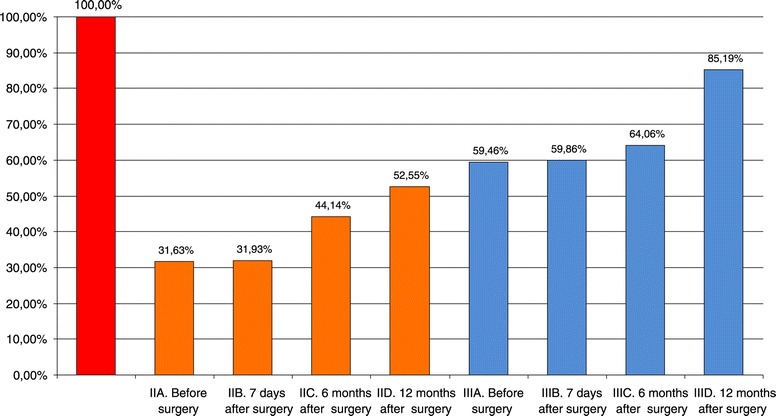
**Lipid index (%) in each group of patients compared with the control group.**

The relationship of the percentage of this parameter in both groups compared with the control group is presented in Figure 1. Before the surgery, both groups had lower lipid indices *WL*, at approximately 31% for Group II and 60% for Group III. After the procedure, a constant upward trend was observed in this parameter, and the data at time D are about 52% for Group II and about 85% for Group III.

## Discussion

Previous studies using the lipid index *WL* have shown its usefulness in monitoring hormone replacement therapy in postmenopausal women.

In the search for useful diagnostic indicators in gynecological oncology, a slight deviation from the norm of lipid parameters was found. However, the application of the lipid index *WL*, which is calculated from HDL, LDL, TG, ApoA1, and ApoB, showed a decrease in this parameter of up to 30% in the serum of patients, as compared with healthy subjects. Further studies have shown differences that depend on the type of gynecological disease.

This information encouraged the authors to conduct further diagnostic tests and monitoring over the 12 months after the operations. Results that are particularly interesting include those for times C and D, where there was an increase in the lipid index *WL*, slowly reaching the values characteristic of healthy women of similar age and BMI. After one year, indices of growth of about 25% for teratoma adultum and about 20% for simple cysts was found, which correlates with the clinical evaluation of the patients.

It appears that further clinical trials on this indicator which also take into account data regarding physical activity, diet, and risk factors for diseases of civilization will allow for the development of a healthy control system based on the observation of lipid metabolism in women.

## Consent

Written informed consent was obtained from the patient for the publication of this report and any accompanying images.

## Abbreviations

BMI: Body mass index
HDL: High-density lipoprotein
LDL: Low-density lipoprotein
TG: Triglyceride
